# Use of vision-based augmented reality to improve student learning of the spine and spinal deformities. An exploratory study

**DOI:** 10.4102/sajp.v77i2.1579

**Published:** 2021-10-29

**Authors:** Gok Kandasamy, Josette Bettany-Saltikov, Julien Cordry, Rob McSherry

**Affiliations:** 1 Department of Allied Health Science, School of Health and Life Sciences, Teesside University, Middlesbrough, United Kingdom; 2 Department of Computer Science, School of Computing, Engineering and Digital Technologies, Teesside University, Middlesbrough, United Kingdom; 3 Department of Nursing, School of Health and Social care, University of Chester, Chester, United Kingdom

**Keywords:** vision-based augmented reality, spinal deformity, spinal biomechanics, cognitive load theory, problem-based learning, pedagogy

## Abstract

**Background:**

Knowledge of anatomy and pathology of the spine together with spinal deformities is integral to several healthcare disciplines. This knowledge is crucial for graduates for assessment and management of patients with spinal problems. Physiotherapy students generally find it difficult to conceptualise the integrity of the structure and function of the spine that affects their acquisition of related physiotherapy skills.

**Objective:**

Our first objective was to introduce and evaluate the use of a Vision-Based Augmented Reality (VBAR) mobile application to teach students the anatomy and accessory movements of the spine. A further objective was to explore student experiences of and engagement with VBAR by conducting a post-lecture survey comparing VBAR to traditional teaching.

**Methods:**

This post-intervention crossover design study included two groups: final year physiotherapy students (*n* = 74) and mean age of 23 (±1.8). The computing department at Teesside University developed the VBAR mobile application. Moreover, a survey adapted from a previously published article was disseminated to students to evaluate their level of understanding following the use of the VBAR application.

**Results:**

The results demonstrated that the median questionnaire scores in students’ perceived level of understanding for the VBAR group were significantly higher than for the traditional teaching group (*p* < 0.05).

**Conclusion:**

The results of this post-intervention survey suggest that the integration of VBAR learning activities results in gains relating to students’ understanding of spinal anatomy, function, pathology and deformities. These findings suggest that VBAR could be an additional teaching tool to support student learning.

**Clinical implications:**

Greater understanding is expected to increase the quality of clinical practice.

## Introduction

Internationally, higher education needs to both acknowledge and embrace the benefits of technologies to enhance not only the teaching and learning environment but also student experience. Indeed, teaching excellence at both undergraduate and postgraduate levels can be enhanced using technological advancements and technologies that simulate scenarios that are difficult to illustrate in a classroom. One rapidly expanding area in the educational setting is ‘mobile learning’. Mobile learning as suggested by Sharples (2000) is a learning process that can be applied across multiple contexts, through both social and content interactions, using personal electronic devices.

The value of mobile learning in higher education is critical for both students and academics on several fronts. Firstly, it is portable and flexible and provides instant access to information. Secondly, the freedom to directly access the information by the students significantly enhances the quality of students’ learning (Fitzgerald et al. 2012). Thirdly, the students’ environment together with the way the technology is used is fundamental to supporting students’ understanding of complex knowledge, academic performance and problem-solving skills of the spine and spinal deformities (Squire & Jan 2007). However, some academics are reluctant to implement mobile devices as a learning and teaching tool, because of a lack of motivation and appropriate training in the use thereof (Boude & Sarmiento 2017; Sánchez-Prieto et al. 2019). In addition to academics’ training, the implementation of mobile learning needs to integrate good pedagogical practice. This should aim for the pedagogical practice to minimise the negative effect of high consumption of mobile hours for leisure purposes and redirect them for more academic use (Alonso-Garcia et al. 2019).

Mobile learning is a form of immersive technology, which has several dimensions contained within it. Two of the main components are Virtual Reality (VR) and Augmented Reality (AR). Virtual Reality is defined as a technology that immerses the user inside a world of computer simulation that offers scenarios and activities that have a high level of realism (Lioce et al. 2015). However, with VR, it is important to acknowledge that whilst immersed in the programme the user cannot view the external real world (Chen & Tsai 2012). In contrast, AR, uses multi-touch interactive screens and simulations contained within mobile technology that significantly enhance the academic delivery of complex learning.

**FIGURE 1:**
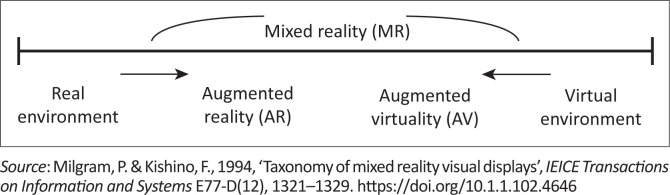
Representation of reality – Virtuality continuum.

Figure 1 illustrates a simple diagram depicting the spectrum of reality. This ranges from the real environment at one end of the spectrum to the virtual environment at the other. Augmented Reality allows the user to view the real world, with virtual objects superimposed upon, combined and overlaying with the real world. This model of AR supplements reality but does not replace it (Andújar, Mejías & Márquez 2011).

**FIGURE 2:**
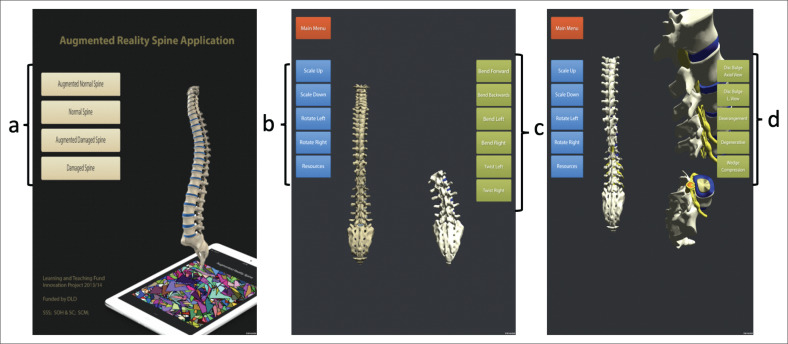
Screenshots of the mobile application depicting (1) the normal structure and function of the spine and (2) various pathologies of the spine. (a) The students were able to choose different modes (from learning about the normal spine to learning different pathologies) within the App. (b) Using the tabs on the left column, students were able to view the spine in all planes. (c) Using the tabs on the right-side column, students were able to explore the biomechanics of different spinal movements in different planes. (d) Using the tabs on the right column, students were also able to explore different clinical conditions of the spine.

**FIGURE 3:**
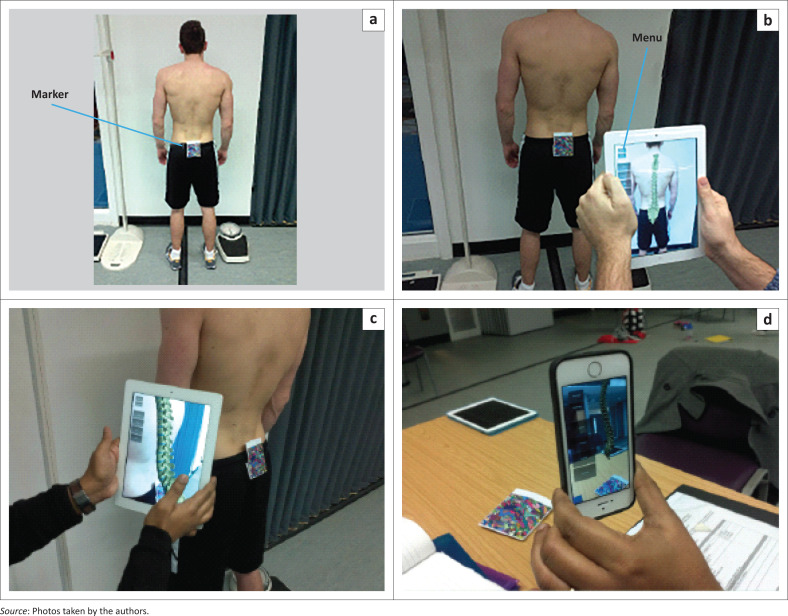
Learning using the vision-based augmented reality mobile application.

Augmented Reality software together with a mobile interface contains ‘context-aware’ technology. Context-aware in this instance refers to the ability of the application to sense the physical environment and adapt its behaviour accordingly (Schilit & Theimer 1994). For example, interactive media can include text, images, audio, video, three-dimension (3D) models and animations as well as multiple-choice or open-ended questions. Images can be viewed through a mobile camera, which enables immersive, collaborative and situated learning experiences. For example, the marker in Figure 3A–D was used in our study to enable students to visualise the 3D spinal models as seen in Figure 2.

A marker-based AR technology used within this context is a physical trigger that when placed on a physical object enables the viewer to experience AR visualisations overlaid on the real object (Butchart 2011). This enables participants to be more ‘immersive’ and to interact with digital information that is superimposed within the physical object or environment (Dunleavy & Dede 2014). Clay (2011) suggests that this combination of immersive interactive learning used together with portable devices provides students with the flexibility of learning at their own pace. This flexibility has, in turn, enhanced students’ experience and acquisition of the performance skills required in real workplace settings, for example, clinical settings (Reznick & Macrae 2006).

Even though there are various examples cited of using AR as a learning and teaching tool in numerous disciplines such as science, mathematics, chemistry, education, biology and engineering, there is less literature on the use of AR in physiotherapy or other healthcare professional literature (Dunleavy & Dede 2014; Kamarainen et al. 2013; Qiu et al. 2021; Reeves et al. 2021; Supruniuk et al. 2020; Tan et al. 2012). Within healthcare higher education, there is an increased emphasis for students to use real-world experiences or tasks to enhance the quality of their learning and its application in clinical practice.

The relevance of using AR approaches to support and enhance student learning is threefold. Firstly, the three-dimensional rotating view and associated imagery of the mechanics of the spine provide students with an instantaneous overview of spinal function. Secondly, in our opinion, this approach facilitates and compliments problem-based learning as well as problem-solving techniques. This is because the student can actually see both the replicas of the ‘normal’ as well as spinal clinical conditions.

Within the field of physiotherapy, the understanding of the segmental movements of the spine is fundamental to successful clinical practice. Knowledge of the numerous coupling (accessory) motions involved within spinal movements is crucial for the physical assessment of patients, the diagnosis of pathological spinal disorders as well as the application of appropriate treatment approaches. However, students generally find it very difficult to visualise and critically analyse the accessory movements (glide and slide) of vertebrae during spinal movements (Noguera, Jiménez & Osuna-Pérez 2013). Furthermore, little is known about the effectiveness of AR for physiotherapy students in a laboratory-based environment and how AR compares to traditional learning within classroom or seminar settings. Therefore, the first objective of our study was to introduce and evaluate the use of a Vision-Based Augmented Reality (VBAR) mobile application to teach students the anatomy and accessory movements of the spine. Further, the second objective was to explore student experiences and engagement of VBAR by conducting a postlecture survey comparing VBAR to traditional teaching. It was hypothesised that the use of VBAR would increase students’ perceived level of understanding of the 3D structure, function and common pathology of the spine in comparison to the traditional method of teaching.

## Methodology

This exploratory study was evaluated using a post-intervention crossover design using two groups (groups 1 and 2) as shown in Table 1. Two tasks were taught to the students. Task (1) involved learning the anatomy of the spine. Task (2) involved learning the pathologies of the spine using both traditional and VBAR learning. The participants were final year BSc physiotherapy students (*n* = 74) with a mean age of 23 (±1.8). The content had not been taught to them before.

**TABLE 1:** A post-intervention crossover study design (*n* = 74).

Variable	Seminar group 1 (*n* = 37)	Seminar group 2 (*n* = 37)
Task 1 (Week 1)	Traditional teaching	Active learning using AR application
Task 2 (Week 3)	Active learning using AR application	Traditional teaching
-	Post survey	-

### Instrumentation

The instrumentation is composed of three components: (1) the development of a VBAR mobile application, (2) a survey and (3) the educational interventions (for both Traditional and VBAR teaching).

#### Development of the vision-based augmented reality mobile application

The VBAR mobile application was developed by the computing department at Teesside University. This comprised an existing 3D spine model and a computer programme called Autodesk Maya. The aforementioned model and programme were used by the computer programmers to design and build both the software as well as the animation. The application that was developed is detailed in Figure 2 and in the following section labelled VBAR-based learning interventions. The application was deployed through the iOS platform and was then accessed by apple mobile devices (iPhone and iPad).

### Evaluation of the exploratory study: A survey of the use of augmented reality as an educational tool

A structured questionnaire was developed to evaluate the use of the VBAR mobile application as an educational tool. The questionnaire comprised nine questions (as seen in Appendix 1). The first eight questions were scored on a 5-point scale (‘strongly agree’, ‘mostly agree; neither agree nor disagree’, ‘mostly disagree’ and ‘strongly disagree’). For the last question (Q9), the students were asked if they had any additional comments. This survey was adapted from the study by Juan et al. (2010). The content validity of the survey was assessed by providing musculoskeletal (MSK) colleagues in the department with a copy of the preliminary survey. They were asked to comment on how well the contents of the questionnaire addressed the dimension of ‘perceived student learning’. Changes to the survey were made following feedback. The reliability of the survey is currently being tested.

#### Educational interventions

Two types of learning (Traditional and Vision Based AR) were compared in our study.

### Traditional learning

Table 2 highlights the learning objectives of the seminars that were used to enhance student learning as identified in the tasks in Table 3. The lecturer provided the students with worksheets that contained 10 questions relating to the material in Table 3. Two-dimensional black and white figures depicting the normal and pathological spine were also included to aid understanding. Students were then asked to search for the answers to these questions from their textbooks and the internet both as individuals and as a group.

**TABLE 2:** The learning objectives in task 1 were focused on understanding the relationship between normal structure and functions of spine in both traditional and Augmented Reality teaching methods.

Number	Description
1	Name the structure and functions of the primary and secondary spinal curves.
2	State the components of a typical vertebrae and how this is different from cervical, thoracic and lumbar spine segments.
3	Name the structures making up transverse process.
4	Identify the joints of lushka (uncovertebral) joints.
5	Describe in detail on the articulation of different segments of spine.
6	What are the factors that influence spinal movement in sagittal, frontal and transverse planes?
7	How would you distinguish lumbar vertebrae?
8	Name and classify the articulating surfaces of the spine?
9	State the orientation of the facet joints, capsules and how is this different from cervical, thoracic and lumbar spine?
10	Explain the structure of the intervertebral disc and its attachment?

**TABLE 3:** The learning objectives in task 2 were focused on understanding the common pathologies of spine in both traditional and Augmented Reality teaching methods.

Number	Description
1	Discuss common spinal injuries in sports.
2	Identify the spinal neural structures formation, courses and its distribution.
3	What are the anatomical structures protect the spinal cord?
4	What are the common sources of spinal pain?
5	Identify the center of pressure in the intervertebral disc and its influence in disc bulge during movement in different planes.
6	What are the different types of spondylolisthesis?
7	Identify the common cause for displacement of vertebrae.
8	What are the possible structural (internal and external) changes takes place during the degeneration of spine?
9	Identify the anatomical structures during spine hyper flexion and extension injuries.
10	State the signs and symptoms of spinal red flags.

### Vision-based augmented reality -based learning

For the VBAR-based learning intervention, the lecturer first demonstrated the mobile application to the students and then clearly highlighted the learning objectives for the tasks (as seen in Table 2 and Table 3). Each student was provided with a mobile device on which the application had been pre-installed. Students then engaged with the developed VBAR mobile application both as individuals and as a group (Figure 3). Students in the AR group used exactly the same course materials (in an electronic format) within the application that the traditional learning group (paper-based) used.

Figure 3 demonstrates an example of a student exploring the VBAR mobile learning material in the seminar to learn the core competencies in task 1. This learner used the mobile phone or tablet to point to a specific marker (trigger) (1), which was placed on the posterior side of the pelvis of a human model. The embedded camera in the mobile device automatically generates and superimposes AR 3D learning material (e.g. the structure of the spine, the normal and abnormal functions of the spine) (2). The images and the information that were generated are shown on the screen of the mobile phone or tablet (3 and 4).

### Procedure

Two seminar groups (1 and 2) from the ‘spine’ and ‘spinal pathologies’ modules were selected to participate in our study. Concerning Table 1, in week 1, group 1 was taught one of the core competencies (task 1 – Spinal Anatomy) using the traditional method of teaching whilst the other group was taught using the AR application. In week 3, the teaching methods for the two groups were crossed over. The second core competency (task 2 spinal pathology) was taught using the VBAR application for seminar group 1 and traditional teaching for seminar group 2. In week 4, all students were asked to fill out the survey to measure their perceived level of understanding of both the normal and the pathology of the spine.

All the learning material was delivered by the same two lecturers. They were also involved in collecting data in week 4. The choice of the lecturer was based on logistical considerations rather than lecturer interest as well as the teaching experiences or propensity for the use of technology. In this experiment, the teaching content was standardised by both lecturers delivering identical content in the same format (refer to Table 2 and Table 3).

### Data analysis

The statistical analysis was conducted using SPSS (Version 21) software. The medians and interquartile range for each question in the questionnaire were analysed using descriptive statistics. The Mann–Whitney U test was used to compare the learning experiences between the traditional and VBAR learning groups.

### Ethical considerations

Our study obtained ethical clearance from the Research Ethics Committee of Teesside University, United Kingdom on 12 February 2014. No reference number was assigned at the time.

## Results

We hypothesised that the use of VBAR mobile application would increase students’ perceived level of understanding with the 3D structure, function and common pathology of the spine in comparison to the traditional method of teaching. A post-survey was carried out to evaluate outcomes associated with the students’ perceived level of understanding with the VBAR mobile application. We quantitatively measured (1) the students’ perceived level of understanding (based on questions Q1, Q2, Q5 and Q6) and (2) the students’ level of engagement (through questions Q3, Q4, Q7 and Q8).

### Comparison of the level of understanding between traditional and vision-based augmented reality learning

Student’s level of understanding was assessed, in Question 1, 89% of the students strongly and mostly agreed that the VBAR helped them understand the normal structure and function of the spine in comparison to 70% from the traditional teaching group. The Mann–Whitney U test result demonstrated a statistically significant difference between the two groups (*p* = 0.005) (Table 4, Q1). The students agreed that the main advantage of using VBAR in spinal anatomy was that it allowed them to actively interact with a 3D spine model in all planes instead of trying to visualise three-dimensional figures and pictures from a two-dimensional picture.

**TABLE 4:** Comparison of students learning experience between traditional and vision-based augmented reality learning group after tasks 1 and 2 (*n* = 74).

A Survey	Traditional learning group (*n* = 74)		AR learning group (*n* = 74)		*Z*	*p* (1-tailed)*
	Median	IQ range	Median	IQ range		
Q1. Teaching session helped me to understand the normal structure and function of spine	4	1.5	5	1	−2.58	0.005*
Q2. I clearly understand the Arthro/Osteo kinematics of spine	4	1	5	1	−2.51	0.006*
Q3. This session made me realise that I had some ideas/misconception about the function and movement of spine	2	11	3	2	−2.39	0.008*
Q4. Session on the normal spine helped me to prepare for the academic and clinical assessment	4	1.5	4	1	−1.65	0.049*
Q5. The teaching session helped me to understand the common pathologies and sources of Low Back Pain	5	1	4	1	−3.67	0.001*
Q6. I clearly understand the structure and function of intervertebral disc.	5	1	4	2	−3.27	0.001*
Q7. This session made me realise that I had some ideas/misconception about the spinal neuro anatomy	3	2	2	1	−2.66	0.004*
Q8. Session on the pathologies of spine helped me to prepare for the academic and clinical assessment	4	1	4	1	−2.27	0.010*

* , statistically significant.

In Question 2, ‘learning the movements of the spine’, 97% of students agreed and mostly agreed that the use of the VBAR application increased their perceived understanding compared to 82% of the students receiving traditional teaching. This difference was statistically significant at *p* = 0.006 (Table 4, Q2).

Under task 2, one group of students already had working experience of using the VBAR application whilst the other group was experiencing it for the first time. In this task, 91% of students agreed that the VBAR application helped them to gain knowledge on common pathologies of the spine, the sources of low back pain as well as the pathologies of intervertebral discs when compared to 63% in the traditional learning group. This also showed a statistically significant difference between the groups (Table 4, Q5 [*p* = 0.001] and Q6 [*p* = 0.001]). Our findings suggest that the inquiry-driven active learning approach using the VBAR application appears to increase physiotherapy students’ perceived level of understanding and scientific thinking on the structure, functions and common pathologies of the spine.

### Engagement and experience

This section discusses the results of using the VBAR application for improving student engagement when learning complex issues. Moreover, we sought to explore students’ experiences of VBAR-based learning in comparison to traditional learning. In Questions 3 and 7, 33% of students agreed that using the VBAR application had helped them realise that they had a number of misconceptions about the function and the movement of the spine and neuroanatomy in comparison to 15% – 18% in the traditional learning group. Results demonstrated between the two groups showed a statistically significant difference between the two groups (Table 4, Q3 [*p* = 0.008] and Q7 [*p* = 0.004]). With regard to the students’ experiences on which mode of teaching best prepared them for their academic and clinical assessment skills, 83% of students agreed that the VBAR helped them more in comparison to 67% of students in the traditional learning group. For question 4, there was only a marginal difference between the two groups with *p* = 0.049, the overall comparison between the groups, demonstrated a significant difference between the groups (Table 4, Q4 [*p* = 0.049] and Q8 [*p* = 0.01]).

Out of all the students who were surveyed 75% agreed that the VBAR application was a powerful tool that helped them to work both individually as well as in a small group. Along with the seminar sessions, most of the students wanted to use this VBAR application on their personal mobile devices to enable them to learn in their own time. In conclusion, 92% of students agreed that the VBAR application was easy to use.

In questions (Q4 and Q8) relating to whether the VBAR application or traditional learning helped students to prepare for their academic and clinical assessment, the medians for both groups were similar. The median score for both tasks showed an Inter Quartile Range (IQR) spanning 1–1.5. Several students offered written feedback to corroborate their responses to some questions as indicated below:

Learning spinal movements by using the Spine AR APP in the PBL is most thought provoking and motivating. I found that I have learned more and retained more in these sessions.

[*T*]he app was great, and was very beneficial to prepare for the spinal assessment and mobilization exam.

[*I*]t has given me more understanding of the pathology as it is hard to visualize.

## Discussion

In summary, our exploratory study sought to examine two hypotheses as stated at the beginning of our study. The results of our survey together with the written feedback indicated that the majority of students found that the VBAR mobile application enhanced their perceived understanding of the anatomy, function and pathology of the spine. We believe that this was achieved by students showing greater engagement with the 3D visualisation and interaction with the virtual spine. Twice as many students agreed that using the AR application was more helpful than traditional learning in clarifying any previous misconceptions that they had regarding the functional movement and neuroanatomy of the spine. The qualitative comments also indicated that students found the VBAR application was easy to use with instructions being clear and easy to understand.

Furthermore, we demonstrated that an inquiry-based learning approach used to engage students in problem-solving and experiential learning within the VBAR application improved students’ perceived subjective understanding of the learning content. Traditionally, spinal anatomy and its function have predominantly been taught using tactile objects such as physical 3D spine models and 2D images. We used inquiry-based learning to engage in problem-solving and experiential learning within the VBAR application, and this improved students’ understanding. Students agreed that the main advantage of using AR in spinal anatomy and pathology was the possibility of interacting with a virtual 3D spine structure instead of imagining the final structure through two-dimensional paper-based figures and pictures. The blending of e-learning contents together with the use of portable devices in classroom teaching offers students the flexibility of mastering core skills at their own pace. Using VBAR also enhanced students’ experiences and acquisition of performance skills that are required in real workplace settings (Clay 2011).

Previously, researchers have attempted to use AR in higher education settings. In the field of biosciences, AR enabled students to manipulate an object in 3D space, interactively exploring protein to protein interactions. Authors have found that this type of learning stimulates peer-to-peer discussions, thereby facilitating dialogic learning (Beltrame et al. 2017; García-Carrión et al. 2020). Similarly, Fombona, Pascual-Sevillana and Gonzalez-Videgaray (2017), in their study, found that an AR teaching approach promotes greater performance in student learning. According to them, this is linked to students’ enhanced creative thinking, motivational and recreational potential and the power of the immersive sense of experience.

In contrast, Cabero-Almenara et al. (2019) carried out a systematic review on the use of technology in higher educational setting, the summary of the findings are as follows: AR facilitates the understanding of complex phenomena, promotes contextualisation and enrichment of information, adapts to different types of intelligence, offers students the ability to interact by manipulating real objects, facilitates the development of a constructivist teaching or learning methodology, promotes the development of graphic skills through the perception of spatial content and 3D objects, it favors learning through practice (experiential learning) and increases motivation with very positive values of satisfaction, as well as improving academic results.

In the same way, our study also suggests that VBAR learning can act as an effective pedagogical tool to support student-centered learning, critical thinking and problem-solving skills. Conversely, Radu (2014) highlighted issues related to AR educational experiences, highlighting that the predominance of students’ ‘tunnel’ attention (tendency to focus on a single point of view), additional cognitive load, usability difficulties, inefficient integration in the classroom and student learning differences are appreciated. Although there are recent studies on projection-based AR in the human body (Ferdous et al. 2019; Hoang et al. 2017), the main limitation is this is more of a laboratory-based delivery of learning material. Because of the lack of studies using mobile AR specifically in physiotherapy students, our findings could not be directly compared with other studies.

Our study has shown that engagement with the VBAR application appears to be superior to traditional learning for helping students understand complex issues and prepare them for academic and clinical assessment (Q3 and Q7). With regard to the understanding of spinal anatomy, movements and pathology of the spine, most students agreed that VBAR was a powerful tool; the VBAR application helped them solve problems both individually as well as in a group. Furthermore, the use of VBAR through handheld technologies increased the ability of students to engage in interactive learning.

From the student comments, it was apparent that the AR app also improved the modes of communication amongst students. For example, the AR app enhanced students’ participation in meaningful scientific discussions and peer discussions. Furthermore, the findings also mirrored those of previous studies on student engagement with the use of AR technology in learning (Dunleavy & Dede 2014; Tang et al. 2020). This can be seen in a study by Tang et al. (2020) where they reported that technology-mediated narratives, as well as interactive, situated, collaborative problem-solving, afforded within AR simulation, were highly engaging in learning in higher education students.

The students’ qualitative comments further suggested that the VBAR intervention resulted in substantial student motivation as seen below. Our results are similar to the findings from Dede (2008), Dede and Richards (2012) where they demonstrated that the use of AR application within classroom teaching fosters increased motivation as well as increased learning:

I really enjoyed the seminar. Learning spinal movements by using the Spine AR APP in PBL is most thought provoking and motivating. I found that I have learned more and retained more in these sessions.

Klopfer and Squire (2008) as well as other authors from diverse disciplines report that both students and teachers report high engagement in education as a result, not only of using handheld mobile devices but also by adopting and being involved in role-playing games. This significantly enhances students’ active involvement in solving authentic problems and engagement in learning complex tasks (Iatsyshyn et al. 2020; Sáez-López et al. 2020; Yu & Conway 2012).

In other questions (Q4 and Q8), both the VBAR and the traditional group had similar median scores. The scores for both types of learning were high, indicating a high level of engagement. The qualitative written feedback received from the large majority of students demonstrated that VBAR helped them prepare, both for academic work as well as their clinical reasoning skills. AR had an impact on both the students’ experience of learning as well as on their preparation for their assessment and was highly praised by most of the students. Some of the comments received can be seen below:

the app was great, and was very beneficial to prepare for the spinal assessment and mobilization exam.

it has given me more understanding of the pathology as it is hard to visualize.

Our results reinforce Cochrane and Bateman (2010) notion that: ‘the pedagogical integration of mobile learning into a course or curriculum requires a paradigm shift on behalf of the lecturers involved, and this takes significant time’. These results demonstrate that an AR interactive learning environment enhances student-learning interest and promotes the learners’ level of engagement in learning complex issues.

## Limitations

The limitations of our study include the following:

- As stated above, the survey used was adopted from the study by Juan et al. (2010). The authors plan to test the reliability of this survey within the higher education setting soon.
- This was an initial study primarily conducted to explore students’ perceptions of the use of VBAR for learning. The authors acknowledge that the study design was weakened by baseline measurements not being undertaken.

## Conclusion and future work

The results of our post-intervention survey suggest that the integration of VBAR learning activities results in a number of learning gains relating to students’ perceived understanding of spinal anatomy, functions, pathology and deformities. Our findings further suggest that VBAR learning through student engagement within the classroom environment may improve healthcare students’ understanding of complex issues. Flexible learning through handheld mobile devices suggests that VBAR allows students to screen capture and record a video of their learning, which can be used as video notes for future reference and revision (Perry et al. 2008). This type of VBAR learning through student interactions in group work appears to facilitate enhanced communication between students and their peers (Tang et al. 2020). The findings from our exploratory study suggest that physiotherapy students subjectively perceived that using VBAR aided them in having a better understanding and learning of the course content compared to the traditional learning group.

Our study has only focused on the immediate perceived learning effects of VBAR. We intend to evaluate the longer-term learning effects of using VBAR within a Cognitive Load Theory (CLT) approach.

## References

[CIT0001] Alonso-Garcia, S., Aznar-Diaz, I., Caceres-Reche, M.-P., Trujillo-Torres, J.-M. & Romero-Rodriguez, J.-M., 2019, ‘Systematic review of good teaching practices with ICT in Spanish Higher Education. Trends and challenges for sustainability’, *Sustainability* 11(24), 7150. 10.3390/su11247150

[CIT0002] Andújar, J.M., Mejías, A. & Márquez, M.A., 2011, ‘Augmented reality for the improvement of remote laboratories: An augmented remote laboratory’, *IEEE Transactions on Education* 54(3), 492–500. 10.1109/TE.2010.2085047

[CIT0003] Beltrame, E.D.V., Tyrwhitt-Drake, J., Roy, I., Shalaby, R., Suckale, J. & Krummel, D.P., 2017, ‘3D printing of biomolecular models for research and pedagogy’, *Journal of Visualized Experiments: JoVE* 121, 55427. 10.3791/55427 PMC5408980 28362403

[CIT0004] Boude, F.O.R. & Sarmiento, A.J., 2017, ‘El reto de formar a profesores universitarios para integrar el aprendizaje móvil’, *Educación Médica Superior* 31(1), 61–77.

[CIT0005] Butchart, B., 2011, *Augmented reality for smartphones*, viewed 19 June 2017, from http://Observatory.Jisc.Ac.Uk/Docs/AR_Smartphones.Pdf.

[CIT0006] Cabero-Almenara, J., Barroso-Osuna, J., Llorente-Cejudo, C. & Fernández Martínez, M.M., 2019, ‘Educational uses of augmented reality (AR): Experiences in educational science’, *Sustainability* 11(18), 4990. 10.3390/su11184990

[CIT0007] Chen, C.-M. & Tsai, Y.-N., 2012, ‘Interactive augmented reality system for enhancing library instruction in elementary schools’, *Computers & Education* 59(2), 638–652. 10.1016/j.compedu.2012.03.001

[CIT0008] Clay, C.A., 2011, ‘Exploring the use of mobile technologies for the acquisition of clinical skills’, *Nurse Education Today* 31(6), 582–586. 10.1016/j.nedt.2010.10.011 21112132

[CIT0009] Cochrane, T. & Bateman, R., 2010, ‘Smartphones give you wings: Pedagogical affordances of mobile Web 2.0’, *Australasian Journal of Educational Technology* 26(1), 1–14. 10.14742/ajet.1098

[CIT0010] Dede, C., 2008, ‘Theoretical perspectives influencing the use of information technology in teaching and learning,’ in *International handbook of information technology in primary and secondary education*, Springer, Boston, MA, pp. 43–62.

[CIT0011] Dede, C., & Richards, J., 2012, *Digital teaching platforms: Customizing classroom learning for each student*, Teachers College Press. https://books.google.co.uk/books?hl=en&lr=&id=Vh5hAgAAQBAJ&oi=fnd&pg=PR5&ots=bNrrER_jdQ&sig=Me9ZVyn3Z6S2-6Rg5ZmPqxVAppA&redir_esc=y#v=onepage&q&f=false

[CIT0012] Dunleavy, M. & Dede, C., 2014, ‘Augmented Reality Teaching and Learning’, in J. Spector, M. Merrill, J. Elen, M. Bishop (eds.), *Handbook of Research on Educational Communications and Technology*, Springer, New York, NY. 10.1007/978-1-4614-3185-5_59

[CIT0013] Ferdous, H.S., Hoang, T., Joukhadar, Z., Reinoso, M.N., Vetere, F., Kelly, D. et al., 2019, ‘“What’s happening at that hip?” Evaluating an on-body projection based augmented reality system for physiotherapy classroom’, in Proceedings of the 2019 CHI Conference on Human Factors in Computing Systems, Association for Computing Machinery, New York, NY, USA, Paper 234, pp. 1–12. 10.1145/3290605.3300464

[CIT0014] FitzGerald, E., Adams, A., Ferguson, R., Gaved, M., Mor, Y. & Thomas, R., 2012, ‘Augmented reality and mobile learning: the state of the art’, in *CEUR Workshop Proceedings*, 955, pp. 62–69. http://ceur-ws.org/Vol-955/papers/paper_49.pdf

[CIT0015] Fombona, J., Pascual-Sevillana, Á. & Gonzalez-Videgaray, M., 2017, ‘M-learning and augmented reality: A review of the scientific literature on the WoS Repository’, *Comunicar. Media Education Research Journal* 25(2), 63–72. 10.3916/C52-2017-06

[CIT0016] García-Carrión, R., López De Aguileta, G., Padrós, M. & Ramis-Salas, M., 2020, ‘Implications for social impact of dialogic teaching and learning’, *Frontiers in Psychology* 11, 140. 10.3389/fpsyg.2020.00140 32116941 PMC7012899

[CIT0017] Hoang, T., Reinoso, M., Joukhadar, Z., Vetere, F. & Kelly, D., 2017, ‘Augmented studio: Projection mapping on moving body for physiotherapy education’, *Proceedings of the 2017 CHI Conference on Human Factors in Computing Systems*, pp. 1419–1430. 10.1145/3025453.3025860

[CIT0018] Iatsyshyn, A., Kovach, V., Romanenko, Y., Deinega, I., Iatsyshyn, A., Popov, O. et al., 2020, ‘Application of augmented reality technologies for preparation of specialists of new technological era’, in *CEUR Workshop Proceedings*, pp. 181–200. http://ds.knu.edu.ua/jspui/handle/123456789/2184

[CIT0019] Juan, C.M., Llop, E. & Abad, F., 2010, ‘Learning words using augmented reality. Advanced Learning Technologies (ICALT)’, in 2010 IEEE 10th International Conference on, pp. 422–426.

[CIT0020] Kamarainen, A.M., Metcalf, S., Grotzer, T., Browne, A., Mazzuca, D., Tutwiler, M.S. et al., 2013, ‘EcoMOBILE: Integrating augmented reality and probeware with environmental education field trips’, *Computers & Education* 440, 1–12. 10.1016/j.compedu.2013.02.018

[CIT0021] Klopfer, E. & Squire, K., 2008, ‘Environmental detectives – The development of an augmented reality platform for environmental simulations’, *Educational Technology Research and Development* 56, 203–228. 10.1007/s11423-007-9037-6

[CIT0022] Lioce, L., Meakim, C.H., Fey, M.K., Chmil, J.V., Mariani, B. & Alinier, G., 2015, ‘Standards of best practice: Simulation standard IX: Simulation design’, *Clinical Simulation in Nursing* 11(6), 309–315. 10.1016/j.ecns.2015.03.005

[CIT0023] Milgram, P. & Kishino, F., 1994, ‘Taxonomy of mixed reality visual displays’, *IEICE Transactions on Information and Systems* E77-D(12), 1321–1329. https://doi.org/10.1.1.102.4646

[CIT0024] Noguera, J.M., Jiménez, J.J. & Osuna-Pérez, M.C., 2013, ‘Development and evaluation of a 3D mobile application for learning manual therapy in the physiotherapy laboratory’, *Computers & Education* 69, 96–108. 10.1016/j.compedu.2013.07.007

[CIT0025] Perry, J., Klopfer, E., Norton, M., Sutch, D., Sandford, R. & Facer, K., 2008, ‘AR gone wild: Two approaches to using augmented reality learning games in Zoos’, *Proceedings of the 8th International Conference on International Conference for the Learning Sciences* 3, 322–329.

[CIT0026] Qiu, X.Y., Chiu, C.K., Zhao, L.L., Sun, C.F. and Chen, S.J., 2021, ‘Trends in VR/AR technology-supporting language learning from 2008 to 2019: a research perspective’, *Interactive Learning Environments*, 1–24.

[CIT0027] Radu, I., 2014, ‘Augmented reality in education: A meta-review and cross-media analysis’, *Personal and Ubiquitous Computing* 18(6), 1533–1543. 10.1007/s00779-013-0747-y

[CIT0028] Reeves, L.E., Bolton, E., Bulpitt, M., Scott, A., Tomey, I., Gates, M. & Baldock, R.A., 2021, ‘Use of augmented reality (AR) to aid bioscience education and enrich student experience’, *Research in Learning Technolog y*, 29, 2572.

[CIT0029] Reznick, R.K. & MacRae, H., 2006, ‘Teaching surgical skills – Changes in the wind’, *The New England Journal of Medicine* 355(25), 2664–2669. 10.1056/NEJMra054785 17182991

[CIT0030] Sáez-López, J.M., Cózar-Gutiérrez, R., González-Calero, J.A. & Gómez Carrasco, C.J., 2020, ‘Augmented reality in higher education: An evaluation program in initial teacher training’, *Education Sciences* 10(2), 26. 10.3390/educsci10020026

[CIT0031] Sánchez-Prieto, J.C., Huang, F., Olmos-Migueláñez, S., García-Peñalvo, F.J. & Teo, T., 2019, ‘Exploring the unknown: The effect of resistance to change and attachment on mobile adoption among secondary pre-service teachers’, *British Journal of Educational Technology* 50(5), 2433–2449. 10.1111/bjet.12822

[CIT0032] Schilit, B.N. & Theimer, M.M., 1994, ‘Disseminating active map information to mobile hosts’, *IEEE Network* 8(5), 22–32. 10.1111/bjet.12822

[CIT0033] Sharples, M., 2000, ‘The design of personal mobile technologies for lifelong learning’, *Computers & Education* 34(3–4), 177–193. 10.1016/S0360-1315(99)00044-5

[CIT0034] Squire, K.D. & Jan, M., 2007, ‘Mad city mystery: Developing scientific argumentation skills with a place-based augmented reality game on handheld computers’, *Journal of Science Education and Technology* 16(1), 5–29. 10.1007/s10956-006-9037-z

[CIT0035] Supruniuk, K., Andrunyk, V. & Chyrun, L., 2020. AR Interface for Teaching Students with Special Needs. In COLINS (pp. 1295–1308).

[CIT0036] Tan, T., Lin, M., Chu, Y. & Liu, T., 2012, ‘Educational affordances of a ubiquitous learning environment in a natural science course’, *Educational Technology & Society* 15, 206–219.

[CIT0037] Tang, Y.M., Au, K.M., Lau, H.C.W., Ho, G.T.S. & Wu, C.H., 2020, ‘Evaluating the effectiveness of learning design with mixed reality (MR) in higher education’, *Virtual Reality* 24(4), 797–807. 10.1007/s10055-020-00427-9

[CIT0038] Yu, F. & Conway, A.R., 2012, ‘Mobile/smartphone use in higher education’, *Proceedings of the 2012 Southwest Decision Sciences Institute*, pp. 831–839. http://www.swdsi.org/swdsi2012/proceedings_2012/papers/papers/pa144.pdf

